# Review of Terahertz Pulsed Imaging for Pharmaceutical Film Coating Analysis

**DOI:** 10.3390/s20051441

**Published:** 2020-03-06

**Authors:** Décio Alves-Lima, Jun Song, Xiaoran Li, Alessia Portieri, Yaochun Shen, J. Axel Zeitler, Hungyen Lin

**Affiliations:** 1 Department of Engineering, Lancaster University, Lancaster LA1 4YW, UK; d.alvesdelima@lancaster.ac.uk (D.A.-L.); songjun@njfu.edu.cn (J.S.); x.li59@lancaster.ac.uk (X.L.); 2 Department of Information Science, Nanjing Forestry University, Nanjing 210037, Jiangsu, China; 3 TeraView Ltd., 1, Enterprise Cambridge Research Park, Cambridge CB25 9PD, UK; Alessia.Portieri@teraview.com; 4 Department of Electrical Engineering and Electronics, University of Liverpool, Liverpool L69 3GJ, UK; Y.C.Shen@liverpool.ac.uk; 5 Department of Chemical Engineering and Biotechnology, University of Cambridge, Philippa Fawcett Drive, Cambridge CB3 0AS, UK; jaz22@cam.ac.uk

**Keywords:** film coatings, TPI, terahertz, characterization

## Abstract

Terahertz pulsed imaging (TPI) was introduced approximately fifteen years ago and has attracted a lot of interest in the pharmaceutical industry as a fast, non-destructive modality for quantifying film coatings on pharmaceutical dosage forms. In this topical review, we look back at the use of TPI for analysing pharmaceutical film coatings, highlighting the main contributions made and outlining the key challenges ahead.

## 1. Introduction

In pharmaceutical manufacturing, film coating on solid oral dosage forms, especially for tablets, is typically performed as the last steps of secondary manufacturing value chain. Coatings are usually applied to cores such as tablets or pellets and in some cases small particles such as crystals. In order to facilitate film formation and processing, coating formulations typically contain polymers and additives such as a plasticizer, anti-adherents, surfactants and colorants [1]. The process of film coating is widely used to ensure colour uniformity, light protection and taste masking of the dosage forms. Functional coating can be used to mask the taste or smell of a product, to protect the active pharmaceutical ingredient (API) against the acidic environment of the stomach or the gastric mucosa against an aggressive API, and to prolong API release. Active coatings contain an API in the coat. They are applied to realise different fixed dose combinations and to prevent interaction of different drugs or to combine different release behaviour in one single solid dosage form. Table 1 provides an overview of different functional coatings.

Coating thickness and integrity are important especially for functional coatings. A minimum thickness and the absence of cracks are required to ensure gastro resistance of a dosage form. When the coating is designed to ensure targeted release profile/rate, coating thickness determines the drug dissolution rate. In active coatings, the amount of API in the coating directly correlates to the coating thickness when a uniform distribution on the core and a constant density of the film layer are achieved. Dosage forms with a targeted release profile are very sensitive to cracks and other imperfections. Such defects in the coating can lead to dose dumping, a spontaneous release of the drug leading to possible toxic side effects. As the coating process is stochastic in nature, coating thickness of the final product will inevitably follow a distribution and therefore, coating uniformity is also important. One needs to distinguish between intra-tablet and the inter-tablet coating uniformity. The former refers to the coating variation between the tablet faces and the band on a tablet, while the latter refers to the variations between representative coatings across the population of tablets [1,2,3,4].

The tablet coating process used is commonly a batch process using a perforated pan-coater. Although such a unit operation has been performed for many decades, initially starting out as sugar coating, there is still a lack of process understanding and control to ensure productions of coatings with low coating variability and imperfections under stringent time requirements. Developments in process analytical technology (PAT) have been made for assessing coatings beyond traditional gravimetric analysis and extensive coverages on the subject matter have been reviewed previously [2,3,4,5]. Of particular interest here is terahertz pulsed imaging (TPI), which was first introduced approximately fifteen years ago and has received a rapid growth in the level of scientific interest ever since. In this review we will look back on the use of TPI, pertaining to pharmaceutical coatings, highlighting the main findings, as well as the challenges ahead.

## 2. Terahertz Pulsed Imaging

### 2.1. Instrumentation

**Figure 1:**
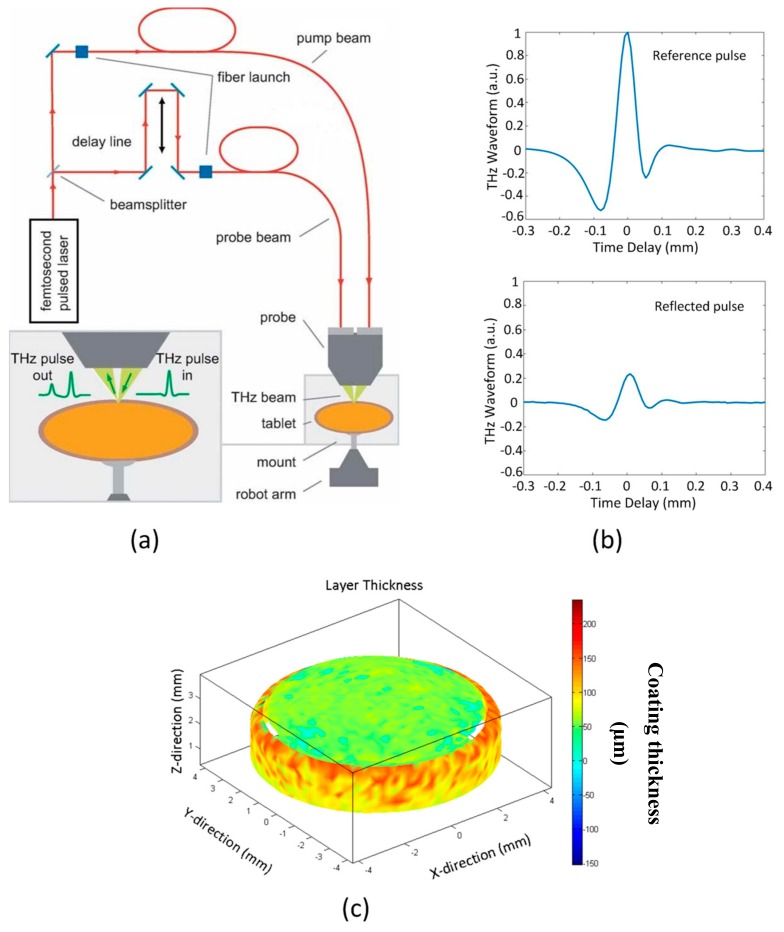
(**a**) Schematic of terahertz pulsed imaging (TPI). (**b**) Raw terahertz waveforms of reference (mirror) and sample (tablet). (**c**) 3D coating thickness image of one face and centre band of a biconvex tablet. The coating layer is thicker on the centre band than on the top face of the tablet. The false colour bar refers to the coating layer thickness, in µm scale. Reprinted from [7] with permission from Elsevier.

TPI Imaga 2000 (TeraView Ltd., Cambridge, UK) was specifically designed for the pharmaceutical industry. The core technology is a reflection terahertz time-domain spectroscopy (THz-TDS) [6,7], which is driven by an ultrafast laser operating at near-infrared (NIR) frequencies (centred at 800 nm). In particular, terahertz pulses are generated by optically exciting a biased photoconductive antenna [8] where the emitted pulses are collimated and focused onto the sample using a silicon lens system at an oblique angle of incidence. The back reflected terahertz pulses are then collected and focused, using the same silicon lens system, onto an unbiased photoconductive antenna for the laser-gated terahertz detection [9]. By rapidly sweeping a mechanical delay line, the transient terahertz electric field can be traced out. Figure 1a shows a schematic of the system.

As most pharmaceutical tablets have curved surfaces, a precise model of the surface shape and curvature of the tablet is required. This model is generated by moving the sample in front of a laser gauge, operating at a wavelength of 670 nm, using the si*x*-axis robot system [7,10]. Once the topology model is acquired, it is then used to keep the sample at the focal position and perpendicular to the terahertz optics. As a result, no sample preparation is needed and most pharmaceutical solid dosage forms with common shapes and surface curvatures can be directly imaged using this instrument.

### 2.2. Coating Thickness Analysis

Imaging of pharmaceutical dosage forms using terahertz radiation is suitable because most excipients do not exhibit any distinct spectral features while being (semi-)transparent to terahertz radiation. This therefore makes it possible to carry out non-destructive imaging at depth. In a standard measurement, the terahertz pulses that are incident on the tablet surface will penetrate through the underlying coatings. At each coating interface or abrupt change in refractive index between drug particles and excipient matrix, a portion of the terahertz pulse reflects back to the detector. This in turn provides the necessary contrast to differentiate between structural layers or subsurface structures within the tablet. Under the assumption that TPI is a linear time-invariant system, the measured waveform is therefore a convolution between sample’s impulse response and TPI’s systems response corrupted by additive white Gaussian noise (AWGN). The impulse response of the sample of interest can therefore be estimated by subtracting away AWGN, taken as the baseline prior measurement, and deconvoluting the measured waveform from the reference waveform, which is a mirror reflection. An example of raw measured waveforms is shown in Figure 1b. In terms of practical implementations, deconvolution is performed by dividing the measured waveform against the reference waveform in the frequency domain. As division would amplify any high frequency noise present in the signal, a customised skewed Gaussian filter is applied as part of the deconvolution process [11]. The result of which, is then transformed back to time-domain for peak-finding. Film coating thickness is determined by the separation of adjacent reflection peaks in the processed signal, given by *d = cΔt/(2n)*, where *d* is the coating thickness, *c* is the speed of light, *Δt* is the peak separation time and *n* is the refractive index of the coating material. It is important to highlight that this method of thickness finding is not adversely affected by imperfect deconvolution settings but requires an abrupt change in refractive index between adjacent layers [12]. The refractive index can be estimated either from strength of the primary reflection using Fresnel’s equations [10], an independent measurement using techniques such as X-ray microtomography (XµCT) [12] or precisely using THz-TDS, for determining the absolute film coating thickness. As a reference, Table 2 lists the refractive indices of common excipients, which were measured using the terahertz pulsed spectrometer spectra 1000 (TeraView Ltd., Cambridge, UK). The majority of the coating analysis routines is implemented in the commercial software package supplied by TeraView.

In order to generate a coating thickness map of a tablet, terahertz waveforms are measured across the entire surface of the tablet thus generating a 3D data cube, where *x* and *y*-axes represent lateral dimensions while *z*-axis represent time-delay or depth. An example of a 3D coating thickness image is displayed in Figure 1c. The lateral resolution is limited by the wavelength of terahertz radiation, for example 150 and 400 µm at 1 and 2.7 THz, respectively [10,13], and the depth resolution is limited by the terahertz pulse duration of <200 fs. The achievable lateral resolution for pharmaceutical tablets is typically between 150–250 µm, at approximately 1.5 to 2.7 THz frequency range, and 30–40 µm for the depth resolution [6,7,10,14,15]. Investigations on improving spatial resolution of a THz-TDS system have been presented elsewhere [16,17,18]. The depth resolution can be significantly improved by numerically fitting the measurement to a terahertz electromagnetic propagation model using values of material’s refractive index and extinction coefficient [19]. In addition to coating thickness, other useful terahertz parameters of interest include (1) terahertz electric field peak strength (TEFPS), which provides information on coating surface roughness and density, given by *TEFPS = 100(S/R)*, where *S* and *R* are the amplitude of reflected terahertz signals from the coating surface and a mirror, respectively [20]; (2) terahertz interface index (TII), which represents physical or chemical composition changes at the interface, given by *TII = 100(I/S)*, where *I* is the amplitude of the reflected terahertz signal from the interface [10]. Despite the wealth of information that can be obtained, it should be noted that where measurement is taken across sample edges, such as embossing and breaking on tablets, or across a very rough surface, it will lead to pronounced scatterings thus yielding unreliable results. However, the effect of scattering at terahertz wavelengths are generally reduced compared to the infrared or visible radiation imaging techniques.

## 3. Pharmaceutical Film Coatings

### 3.1. Off-Line Measurement

#### 3.1.1. Coating Morphology, Density and Drug Release Performance

**Figure 2:**
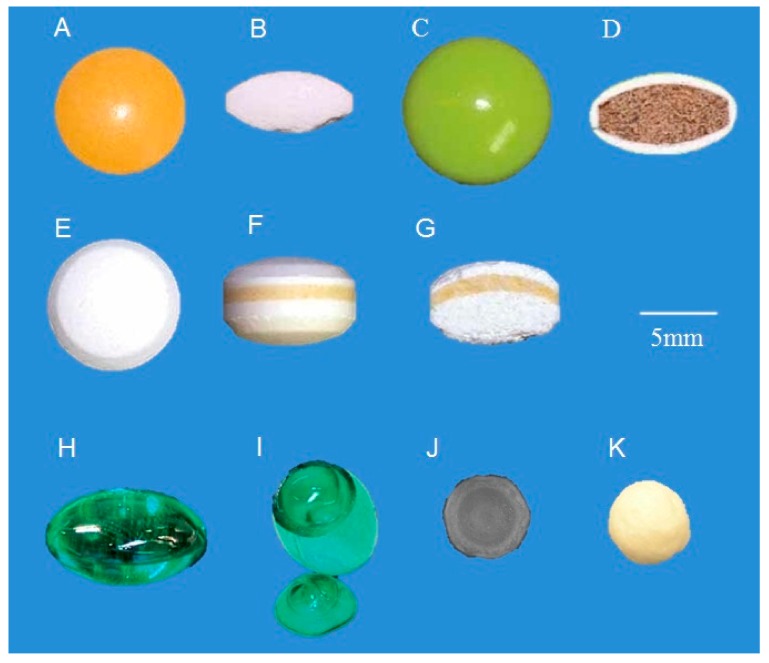
Picture of several solid oral dosage forms analysed with TPI. (**A**) Top and (**B**) cross-section view of an enteric coated tablet; (**C**) top and (**D**) cross-section view of a sugar-coated tablet; (**E**) top, (**F**) side, and (**G**) cross-section view of a tri-layered controlled release tablet; (**H**) side and (**I**) cross-section view of a soft gelatin capsule; (**J**) cross-section and (**K**) top view of a pellet. Reprinted from [7] with permission from Elsevier.

In the pioneering work by [6], TPI was used to analyse coating thickness of commercially available ibuprofen tablets. By raster scanning the terahertz beam across a 1 × 1 mm square section of the tablet at 50 µm steps, TPI was able to resolve different coating layers, measure overall thicknesses, and distinguish single- and multicoated tablets from different manufacturers. These results were in good agreement with the destructive optical microscopy measurements. The success of this work led to the development of TPI for surface imaging of arbitrary shaped tablets automatically [7]. This in turn provided information on the spatial and statistical distribution of coating thickness for a wide range of products such as film-coated tablets, sugar-coated tablets, multi-layered controlled release tablets and soft gelatin capsules (Figure 2). For film coatings that protect the active ingredient against light and moisture, TPI has been applied to assess coating uniformity and density analysis [21].

TPI is used to image delayed-release tablets, where average coating thickness correlated well against dissolution behaviour under simulated gastric pH conditions, in the form of mean dissolution time (MDT) [22]. It was shown that with the increasing coating thickness, dissolution rate decreases but this correlation only existed when the coating thickness over entire tablet surface is taken as opposed to a randomly selected tablet face. In the case of sustained-release tablets, an inverse relationship was found where higher average coating thickness actually resulted in faster dissolution times, while a higher TEFPS value resulted in a longer dissolution time [20]. More importantly, is the correlation between terahertz parameters and product performance during a process scale-up from lab-scale (4 kg) to pilot-scale (20 kg), which can be used to predict the dissolution behaviour as opposed to the traditional weight gain. Extending on this work but with an increasing polymer weight gain at 10% increments, it was further found that while coating layer thickness data are strongly correlated to MDT in both lab and pilot-scales, ultimately coating density (determined by TEFPS) showed a more prominent effect on dissolution during process scale-up [23]. For large-sized sustained-release coated pellet dosage forms (average diameter approximately 6 mm), TPI was also employed to investigate the effects of film coating thickness, drug layer uniformity and the effect of curing on in vitro drug release [24]. Results of the analysis indicated that the terahertz parameters were not indicative of the drug release performance for these pellet dosage forms possibly because of a non-diffusion drug release mechanism through the film coating structure.

The feasibility of TPI for an active coating process was demonstrated in [25]. In the study, a linear correlation was found between the API content, as independently assessed by high performance liquid chromatography (HPLC), and coating thickness in the range between 50 to 500 µm, as assessed by TPI and optical microscopy. An extensive discussion on the relevant parameters, which need to be controlled especially in active coatings so as to not misinterpret the TPI measurements was presented [26]. By comparing the content measurements as measured by an HPLC assay and the TPI coating thickness measurements it was possible to establish an excellent correlation between them. An obvious advantage of TPI for the analysis of active coating processes is the speed and ease of measurement compared to an HPLC content assay: no sample preparation was required; no solvents were used, and each measurement was completed in well under one hour and the sample is intact after TPI measurements thus they can be reused if needed.

In the case of push-pull osmotic systems (PPOS), an osmotic-controlled release technology based on multiple coatings, TPI was successfully applied to investigate the coating characteristics [27], where coating thickness distribution variations all correlated with the drug release kinetics and internal physical changes leading to slower drug release, confirmed by optical microscopy analysis.

In a proof-of-concept study [28], TPI was applied to analyse the inner structure of multiple unit pellet system (MUPS) tablets, an additional controlled release technology where API-containing pellets are present in the coating formulation. TPI was found to be suitable to detect the pellets in the coating structure up to 152 µm depth. By increasing the number of pellets, and comparing with XµCT images, TPI analysis could accurately visualise the number of pellets up to 40% (w/w) pellet amount in MUPS tablet. Undetected pellets were mostly localised around the edges of the tablet, suggesting the presence of edge effects in the terahertz waveforms thus limiting resolution.

#### 3.1.2. Defects Identification

A number of studies have also taken the advantage of TPI to penetrate through the coating structures to identify coating defects, first demonstrated in [29] to detect holes and defects in sustained-release coats. This work has been extended to detecting cracks between layers in bilayer tablets [30] where the adhesion integrity can be quantified based on the TII, which in turn can be correlated to layer separation tendency. TPI can also reveal structural defects in enteric coatings where the combination of coating thickness, coating uniformity, TEFPS and TII information can infer on the physiochemical properties of the coating and acid resistance [31]. The results of which were successfully validated against scanning electron microscopy (SEM), XµCT, acid uptake tests and loss on drying (LOD) test, a measure of water content in sample tablets. TEFPS was previously shown to reveal information on surface density and roughness [20,21] and it was further demonstrated in [32] how TEFPS could additionally provide information on the physical strength of the tablet surface for the estimation of surface defect risk. The ability to resolve air gaps in turn could provide information on adhesion quality between core and coatings in calendered tablets [33]. Finally, TPI was also used for detecting cracks in coatings stored at high temperatures (60–70 °C) [34].

#### 3.1.3. Comparison and Calibration of PAT

The ability of TPI to measure the absolute coating thickness has made it an attractive reference method for comparison and calibration against various PAT. For example, it has been compared against the well-established NIR spectroscopy for coating thickness and uniformity of production-scale pharmaceutical dosage forms [14]. Comparing the two techniques, NIR spectroscopy is an indirect measurement, where coating thickness needs to be correlated with the absorbance that in turn requires multivariate calibration using partial least squares (PLS) for quantitative analysis. TPI, on the other hand, measures coatings directly without calibrations. The two techniques are, however, considered to be complementary where NIR is more accurate for thinner coatings (0–4% weight gain), while TPI is suitable for thicker coatings (4–5% weight gain). Both techniques could analyse inter- and intra-tablet coating uniformity as well as identifying defects [35], with NIR being constrained for only the top and bottom surfaces of biconvex shaped tablets, while TPI is applicable for thicker coating layers [36,37]. TPI additionally can be used for calibrating in-line PAT such as NIR [38] and Raman [39] probes, which when taken together with MDTs, can be used for model development to quantitatively predict dissolution behaviour of sustained-release tablets [15]. In particular, the model was built with data from a batch, and successfully predicted MDTs for another batch produced under similar process conditions, and detect coating thickness variability in a different batch.

**Figure 3:**
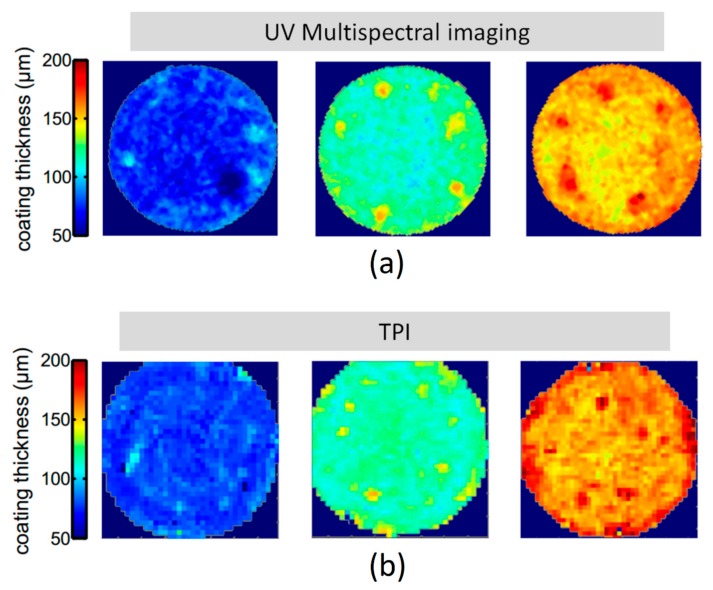
Coating thickness distribution on tablet surface as (**a**) predicted by partial least squares (PLS) using ultraviolet (UV) spectral information and (**b**) measured by TPI. Reprinted from [40] with permission from Elsevier.

In a manner similar to NIR but at a shorter wavelength, the feasibility of multispectral ultraviolet (UV) imaging was performed on theophylline film-coated tablets where TPI was used as the reference measurement [40]. The study showed that even though both methods resulted in a similar coating profiles, as shown in Figure 3, UV imaging like NIR is an indirect measurement, where coating thickness is correlated against reflectance spectra using multivariate PLS models. UV imaging nevertheless can operate at a higher acquisition rate (30 s) and spatial resolution because of a shorter wavelength.

With the emergence of spectral-domain optical coherence tomography (OCT) for coating evaluation, TPI has also been used as the reference where it was found that the two techniques are complementary. In particular, OCT is well suited for thin coatings 10–60 µm, while TPI is suitable for 40 µm and above. This is reinforced in [41], which also showed how coating refractive index at optical frequencies could be extrapolated from the absolute thickness measured using TPI [42]. Ultimately, the upper limit for OCT measurement is bound by the optical properties of the coating formulation [43]. This work is then extended for evaluating intra-tablet coating uniformity [44].

Finally, TPI has also been compared against XµCT to assess the validity of using a constant refractive index across the entire surface of a tablet [12]. Results of the study confirmed the acceptable error of 3–4% for coating layers 25–270 µm thick across the tablet surface.

#### 3.1.4. Process Understanding

**Figure 4:**
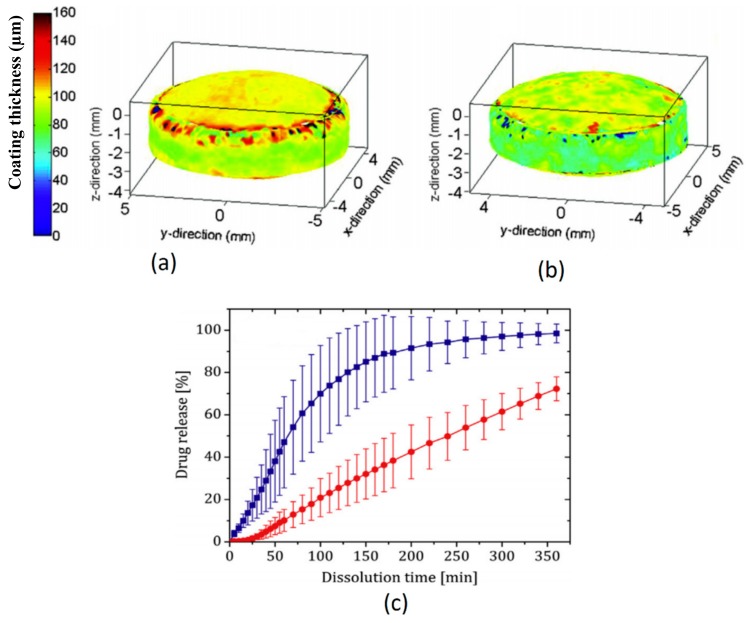
3D coating layer thickness maps of (**a**) fluid bed-coated tablets, (**b**) pan-coated tablets and (**c**) respective dissolution profiles. Thicker, less dense and more uniform fluid bed coating (blue squares) achieved a faster drug release compared to pan-coating (red circles). The false colour bar is in µm scale. Reprinted from [48] with permission from Elsevier.

Understanding the coating unit operation is imperative to improve the product quality and reduce output risks. A high level of intra-tablet and inter-tablet coating uniformity are the desired quality attributes especially for modified-release coatings, where a high level of coating variability can potentially undermine the efficacy of the eventual product. TPI, owing to its relatively high spatial resolution, has shown to be a suitable tool for quantifying active coating thickness uniformity of tablets coated under varying process conditions [45,46]. In particular, using design of experiments covering a wide range of realistic coating process conditions for process parameters such as drum load, drum rotation speed, spray rate, spray pressure and coating duration, TPI was used to identify and optimise the critical process parameters for an active coating process of gastrointestinal therapeutic systems (GITS). The study found that a combination of low drum load, high drum rotation speed and long coating durations are factors that could improve the intra-tablet and inter-tablet uniformity. Even though a low spray rate was shown to be beneficial for inter-tablet coating uniformity, the same setting would be counter-productive in reducing the level of intra-tablet coating uniformity. Other quality attributes, such as surface roughness, tensile strength and risk of cracking can also be determined based on values of TEFPS and TII where tensile strength could be predicted by multivariate analysis using TII for film coated tablets with hydrophilic cores [47]. It was found that inlet air temperature, spray rate and atomised air volume had significant impact on the coating thickness and TII, while no process parameters were demonstrated to have impact on TEFPS. These parameters can also be used to assess the coating performance of different coating equipment, such as a fluid bed or pan-coater [48]. In particular, these parameters exhibited substantial differences for example, centre band thicknesses were substantially thinner than top and bottom surfaces in pan-coated tablets (22.5%) as opposed to fluid bed coated tablets (12.5%). Further, pan-coated tablets showed more prolonged drug dissolution profile (72% in 6 h) compared to fluid bed coated tablets (98% in 6 h), as shown in Figure 4.

With an increasing availability of computational power, numerical simulation are used to understand the film coating process [49,50,51,52,53]. In particular, TPI was used to validate discrete element method (DEM) simulated data on intra-tablet coating thickness distribution where a reasonable agreement with measurement for tablets of different shapes (almond, oval, round and triangular) was observed [52].

### 3.2. In-Line Measurement

The feasibility of TPI as an in-line PAT for future quality control and process understanding was demonstrated for the first time in [54]. The study exploited the fact that moving tablets have a tendency to align themselves facing toward the metallic mesh of the coating drum and therefore by focusing the terahertz pulses from outside the drum into the coater, coating thickness of individual tablets could be measured directly. The fibre-coupled terahertz sensor was externally mounted such that the focus plane was on the inner surface of the metal mesh of the coating pan. The measurements were then automatically processed using an algorithm that selected waveforms representative of off-line reflections from the coated tablets for thickness calculation [55,56]. To validate the in-line acquired thickness measurements, weight gain and thicknesses of randomly selected tablets were taken and compared throughout the process. Compared with the off-line TPI, this demonstration is unique in that it can measure the tablet coating thickness directly with sufficiently fast acquisition rate (up to 120 Hz), obtained only from a single spot of the moving tablets, yielding statistical information on the coating variability of the tablet population inside the coating unit (inter-tablet coating uniformity).

Building on from the proof-of-concept work, is the demonstration on how the in-line TPI could be used to resolve changes to coating thickness in response to changing process conditions [56] in a production-scale process. Notably, the following observations were made: (1) Removal of mixing baffles during the coating process led to coatings with a higher level of thickness variation due to poorer tablet mixing; (2) adding a batch of uncoated tablets during the coating operation resulted in the clear observation of two distinct thickness populations; (3) reducing the spray rate and halting the coating pan during the coating process resulted in a clear reduction thickness growth rate and tablet hit rate, respectively. Even though the process changed made were entirely artificial and extreme in some cases, the study showed the sensitivity of in-line TPI for coating process investigation and monitoring.

**Figure 5:**
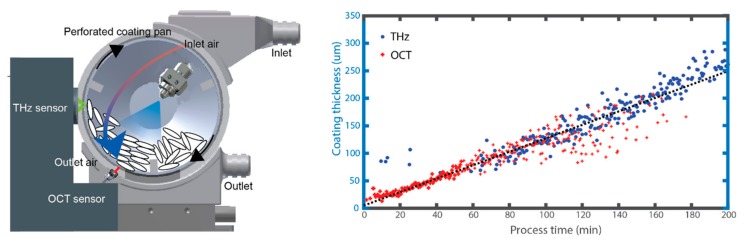
Schematic of the lab-scale tablet coater for performing combined OCT and THz in-line sensing of the pharmaceutical tablet coating process (left). Time average of the coating thickness measured by the respective sensors independently where thin coatings can be resolved by OCT and thicker coatings with TPI. Reprinted from [41] with permission from Elsevier.

As process investigation on the production-scale are wasteful and expensive, a lab-scale coating unit was purposely built, retrofittable to the TPI [41], shown in Figure 5. Uniquely, the coating unit also included provisions for in-line OCT sensing, which as a whole allows coating thicknesses from as low as 20 μm and up to 300 μm and above to be measured and also with different coating formulations. This is shown in Figure 5 with OCT dominating the first 50 min of the coating process followed by TPI thereafter until process end-point. The availability of film coating thickness measurements from individual tablets in turn provided substantially more information on the inter-tablet coating uniformity as well as intra-tablet coating uniformity with OCT. However, as the process was not optimally controlled, this resulted in coated products an increased porosity as evaluated by XμCT and off-line TPI.

To better understand the coating process and explain the observations, numerical simulation using DEM was suggested. As an initial step, the tablet coating process was simulated numerically [57], combined with a ray-tracing technique and validated against in-line TPI measurement of a pre-coated tablets during mixing in a rotating pan. Metrics of validation included the measurement rates and thickness distribution under increasing number of baffles in the mixing pan, where a good agreement was observed. The experimentally validated numerical model in turn could be used to better inform the terahertz measurement such as the measurement location. In addition, the numerical model could be used to fine-tune the settings of the selection criteria as part of the waveform selection algorithm, which presently can only be estimated from off-line measurements alone, such as the strength of the reflection from the buried surface [55]. Uniquely, this study highlighted how in-line, in-process measurements could be used to validate numerical models in a field where off-line measurements taken at process endpoints have been used.

## 4. Conclusions and Future Challenges

Measurements with TPI to date have revealed a wealth of microstructure related information in pharmaceutical dosage forms. Table 3 summarises the main findings on TPI applications for pharmaceutical coating analysis. Compared to competing technologies, TPI is non-destructive, provides an excellent contrast between coating structures despite a similarity in refractive indices between adjacent layers and can penetrate through thick coatings without being affected by scattering losses from pigments and air bubbles in the structure. However, TPI can only resolve coatings greater than 35 µm without using advanced data extraction methods [23], but cannot resolve coating structures on tablet features with sharp edges or in embossings, and cannot fully image through the entire volume of the dosage forms largely due to the optics used [12]. Further, it can only measure coatings on pellets with dimensions >500 µm. For TPI to become widely deployed across pharmaceutical industry in a manner similar to how NIR and Raman spectroscopy has impacted the field, the technology requires faster acquisition times, more reliability especially with laser operational stability and increased affordability. Additionally, advances in signals processing techniques needs to be made such as resolving spectral signatures at depths for 3D chemical mapping and accounting for scattering losses from sharp edges and at depth. At the same time, improvements in waveform selection algorithms are desired in order to harness the many advantages this modality has at an in-line setting. Finally, like any other PAT, the acquired data quickly build up, leading to the necessity of addressing the issue of data management or optimal sampling strategy [46]

## Author Contributions

D.A.-L. and H.L. wrote the manuscript. A.P. provided the data. All authors proofread the manuscript. All authors have read and agreed to the published version of the manuscript.

## Figures and Tables

**Table 1:** Summary of functional coating types and their respective functions [1,2].

Type of Coating	Coating Function
Active	Coating layer contains API
Sustained-release	Allows for predetermined API release rate for an extended time period using water-insoluble polymers
Controlled-release	API release profile is designed to ensure constant drug concentration in the body
Delayed-release	Aims to control the site of drug release, for example oesophagus, intestine (enteric) or colon
Osmotic-controlled	API is released via osmotic pressure with the aid of a semipermeable membrane
Enteric	Prevents dissolution or disintegration in gastric environment by incorporating polymers featuring ionisable groups
Soft gelatin capsule	Gelatin shell with a non-aqueous liquid filling, improves bioavailability of hydrophobic drugs

**Table 2:** Spectral refractive indices of common excipients for pharmaceutical coatings.

Excipient Material	Refractive Index ^a,b^
Acacia spray dried	1.71 ± 0.02
Acesulfame	2.05 ± 0.01
Avicel PH101	1.70 ± 0.03
Avicel PH102	1.77 ±0.03
Avicel PH200	1.67 ± 0.03
Avicel PH302	1.76 ± 0.02
Avicel RC581	1.76 ± 0.01
Calcium carbonate	2.13 ± 0.03
Calcium phosphate	2.50 ± 0.01
Calcium phosphate dibasic anhydrous	3.25 ± 0.05
Carboxymethyl cellulose	1.76 ± 0.03
Carboxymethyl cellulose sodium	1.73 ± 0.01
Carnuba wax	1.77 ± 0.01
Confectioners’ sugar	1.71 ± 0.005
Cornstarch	1.62 ± 0.01
Dextrose	1.74 ± 0.01
Hydroxypropyl cellulose	1.47 ± 0.01
Lactose anhydrous	1.69 ± 0.005
Magnesium hydroxide	1.78 ± 0.01
Magnesium oxide light	1.44 ± 0.005
Methyl paraben	1.66 ± 0.02
Magnesium stearate 1	1.37 ± 0.005
Magnesium stearate 2	1.34 ± 0.005
Magnesium stearate anhydrous	1.34 ± 0.005
Polyvinylpirrolidone K30	1.60 ± 0.01
Povidone	1.56 ± 0.005
Pregelatinised starch	1.65 ± 0.01
Silicon dioxide colloidal	1.26 ± 0.01
Sodium bicarbonate	1.97 ± 0.02
Sodium carbonate	2.01 ± 0.01
Sodium carboxymethyl	1.78 ± 0.01
Sodium lauryl sulphate	1.68 ± 0.01
Sodium starch glycolate	1.81 ± 0.01
Stearic acid	1.53 ± 0.02
Sucrose	1.83 ± 0.01
Sugar tab	1.70 ± 0.01
Tartaric acid	1.85 ± 0.03
Titanium dioxide	2.29 ± 0.02
Xyloitol	1.76 ± 0.03
Xyloitol 300	1.83 ± 0.03

^a^ Refractive indices are an average representative value between 0.45 to 1.8 THz. ^b^ Data presented as average ± standard deviation.

**Table 3:** Summary of TPI application for pharmaceutical coating analysis.

Reference	Benchmark/Supporting Measurement	Materials	Scale	Terahertz Parameters	Additional Information
Fitzgerald et al. (2005) [6]	Optical microscopy	Film-coating	Production scale	Coating thickness	Imaging area side: 1 mm Coating thickness: 320–450 µm
Zeitler et al. (2007) [7]	-	Enteric-coating, sugar-coating, 3-layered controlled release tablets, soft gelatin capsules with liquid filling	Production scale	Coating thickness and distribution	Imaging acquisition time: 20–50 min Coating thickness: 38–2000 µm
Cogdill et al. (2007) [14]	Optical, microscopy, NIR spectroscopy, weight gain	Film-coating	Production scale	Coating thickness and distribution	Imaging acquisition time: 7 min Imaging area radius: 3.5 mm Coating thickness: 30–64 µm
Spencer et al. (2008) [22]	USP dissolution	Delayed release tablets	Production scale	Coating thickness and distribution	Imaging spot size: 100 µm Coating thickness: 60–110 µm
Ho et al. (2008) [20]	Ph. Eur. dissolution, SEM, weight gain	Sustained-release tablets	Lab (4 kg) and pilot scale (20 kg)	Coating thickness and distribution, TEFPS	Coating thickness: 50–300 µm
Ho et al. (2009) [23]	USP dissolution	Sustained-release tablets	Lab (4 kg) and pilot scale (20 kg)	Coating thickness, TEFPS	Imaging acquisition time: 45 min Coating thickness: 38–151 µm
Ho et al. (2009) [24]	SEM, stereo-microscopy imaging, USP dissolution	Sustained-release pellets	Lab scale	Coating thickness and distribution, TEFPS	Imaging area radius: 3 mm Coating thickness: 14–127 µm
Ho et al. (2009) [15]	USP dissolution	Sustained-release tablets	Lab scale (4 kg)	Coating thickness and distribution, TEFPS, TII	Imaging acquisition time: 45 min
Malaterre et al. (2009) [27]	Optical microscopy, USP dissolution	PPOS (osmotic-controlled)	Lab scale	Coating thickness and distribution	Imaging acquisition time: 15 min Coating thickness: 112–268 µm
Maurer et al. (2009) [35]	NIR spectroscopy, weight gain	Film-coating	Production scale	Coating thickness and distribution	Imaging acquisition time: 20–30 min Coating thickness: 22–92.5 µm
Gendre et al. (2011) [38]	In-line NIR sensor, weight gain	Film-coating	Lab scale (4 kg)	Coating thickness and distribution	Number of point measurements: 3200
May et al. (2011) [54]	Weight gain	Film-coating	Production scale (175 kg)	Coating thickness and distribution, inter-tablet coating uniformity	In-line TPI sensor Acquisition rate: 120 Hz Coating thickness: 40–160 µm
Sakamoto et al. (2012) [21]	-	Film-coating	Production scale	Coating thickness and distribution, TEFPS	Imaging acquisition time: 30 min Coating thickness: 40–150 µm
Brock et al. (2012) [25]	HPLC, Optical microscopy	Active-coated PPOS (osmotic controlled)	Lab scale (3 kg)	Coating thickness and distribution	Imaging acquisition time: 20 min Imaging area radius: 3 mm Coating thickness: up to 500 µm
Russe et al. (2012) [12]	XµCT	Film-coating	-	Coating thickness and distribution	Imaging acquisition time: 45 min Coating thickness: 25–270 µm
Müller et al. (2012) [39]	In-line and offline Raman spectroscopy, USP dissolution	Sustained-release tablets	Lab scale (3.5 kg)	Coating thickness and distribution	System bandwidth: 0.06–3 THz Coating thickness: 40–140 µm
Haaser et al. (2013) [48]	SEM, UV-Vis spectrophotometry, weight gain	Delayed-release tablets	Lab scale	Coating thickness and distribution, TEFPS, TII	Imaging acquisition time: 60 min Coating thickness: up to 160 µm
Brock et al. (2013) [45]	-	Active-coated GITS (osmotic controlled)	Pilot scale (~40 kg)	Coating thickness and distribution, intra-tablet coating uniformity	Imaging area radius: 1.5–4 mm Coating thickness: 76–358 µm
Brock et al. (2014) [46]	HPLC	Active-coated GITS (osmotic controlled)	Lab (3–8 kg) and pilot scale (38–43 kg)	Coating thickness, inter-tablet coating uniformity	Imaging area radius: 1.5–4.5 mm Coating thickness: 360–500 µm
Niwa et al. (2014) [31]	Acid uptake, LOD, SEM, XµCT	Enteric-coating	Lab scale	Coating thickness and distribution, TEFPS, TII	Coating thickness: 50–70 µm
Lin et al. (2015) [42]	OCT	Sustained-release tablets	Pilot scale (20 kg)	Coating thickness and distribution, intra-tablet coating uniformity	Imaging spot size: 200 µm Coating thickness: Up to 300 µm
Freireich et al. (2015) [52]	DEM simulations	Film-coating	Lab scale (1 kg)	Coating thickness and distribution	Imaging acquisition time: 120 min Coating thickness: 40–100 µm
Lin et al. (2015) [56]	-	Film-coating	Production scale (175 kg)	Coating thickness, inter-tablet coating uniformity	In-line TPI sensor Acquisition rate: 120 Hz Coating thickness: Up to 300 µm
Dohi et al. (2016) [47]	-	Film-coating with hydrophilic core	Pilot (36 kg) and production scale (330 kg)	Coating thickness and distribution, TEFPS, TII	Imaging acquisition time: 20–30 min Coating thickness: 35–40 µm
Lin et al. (2017) [41]	In-line OCT sensor, weight gain	Sustained-release tablets	Lab scale (300 g)	Coating thickness and distribution, inter-tablet coating uniformity	In-line TPI sensor Acquisition rate: 30 Hz Coating thickness: 20–300 µm
Novikova et al. (2017) [40]	Multispectral UV imaging, weight gain	Film-coating	Lab scale	Coating thickness and distribution	Imaging acquisition time: 15 min Coating thickness: 50–200 µm
Novikova et al. (2018) [28]	XµCT	MUPS (controlled–release)	Lab scale (~400 g)	Coating thickness and distribution	Imaging acquisition time: 25 min Penetration depth: 152 µm
Pei et al. (2018) [57] Lin et al. (2018) [55]	DEM simulations combined with ray tracing	Film–coating	Lab scale	Coating thickness and distribution, inter and intra–tablet coating uniformity	In–line TPI sensor Acquisition rate: 30 Hz Coating thickness: Up to 100 µm
